# Interstitial pneumonia pattern on day 7 chest radiograph predicts bronchopulmonary dysplasia in preterm infants

**DOI:** 10.1186/s12887-017-0881-1

**Published:** 2017-05-15

**Authors:** Hye-Rim Kim, Ji Young Kim, Bo La Yun, Byoungkook Lee, Chang Won Choi, Beyong Il Kim

**Affiliations:** 1 Department of Pediatrics, Bundang CHA Medical Center, CHA University, Seongnam, Republic of Korea; 2 Department of Radiology, Seoul National University Bundang Hospital, Seongnam, Republic of Korea; 3 Department of Pediatrics, Wonju Severance Christian Hospital, Wonju, Republic of Korea; 4 Department of Pediatrics, Seoul National University College of Medicine, Seoul, Republic of Korea; 5 Department of Pediatrics, Seoul National University Bundang Hospital, 82 Gumi-ro 173 Beon-gil, Bundang-gu, Seongnam, 13620 Republic of Korea

**Keywords:** Bronchopulmonary dysplasia, Chest radiograph, Predictor, Interstitial pneumonia

## Abstract

**Background:**

Early identification of infants at higher risk of developing bronchopulmonary dysplasia (BPD) may enable a targeted approach to reduce BPD. We aimed to evaluate the hypothesis that the interstitial pneumonia pattern on the day 7 chest radiograph predicts BPD or death before 36 weeks postmenstrual age (PMA).

**Methods:**

A retrospective cohort study was performed on 336 preterm infants (birth weight < 1500 g and gestational age < 32 postmenstrual weeks) who were admitted to a single tertiary academic center between January 2008 and December 2014. Day 7 chest radiographs were independently reviewed by two pediatric radiologists who were unaware of the clinical information regarding each individual infant.

**Results:**

Data from 304 infants who survived more than 7 days after birth were collected. The interstitial pneumonia pattern on the day 7 chest radiograph was independently associated with BPD or death before 36 weeks PMA (odds ratio [OR] 4.0, 95% confidence interval [CI] 1.1–14.4). The interstitial pneumonia pattern on the day 7 chest radiograph predicted BPD or death with a specificity of 98%. Histologic chorioamnionitis was a preceding factor that was independently associated with the interstitial pneumonia pattern on the day 7 chest radiograph (OR 3.7, 95% CI 1.3–10.3).

**Conclusions:**

The interstitial pneumonia pattern on the day 7 chest radiograph has a high specificity for predicting BPD or death and can be utilized to select high-risk preterm infants who will benefit from potentially preventive interventions against BPD.

**Electronic supplementary material:**

The online version of this article (doi:10.1186/s12887-017-0881-1) contains supplementary material, which is available to authorized users.

## Background

Bronchopulmonary dysplasia (BPD) is a major cause of mortality and morbidity in preterm infants [1]. Neonatal care has improved dramatically over recent decades, and antenatal corticosteroid treatment, surfactant replacement therapy and more sophisticated assisted ventilation have greatly reduced the severity of respiratory morbidity and mortality. Nonetheless, the incidence of BPD has not decreased [2–5]. Approximately 20 to 40% of very low birth weight (VLBW, birth weight < 1500 g) infants and 12 to 13% of preterm infants born before 32 weeks postmenstrual age (PMA) are affected by BPD [6, 7].

Identification of perinatal or early postnatal predictors of BPD may enable us to implement potentially preventive strategies before BPD has been established. Numerous studies have attempted to identify predictors for BPD. Scoring systems for predicting BPD that include gestational age, birth weight, gender, patent ductus arteriosus (PDA), sepsis and mechanical ventilation in regression models have been presented [8]. Laughon et al. [9] developed a predictive model using data from the NICHD Neonatal Research Network Benchmarking Trial. This model incorporated gestational age, birth weight, race, ethnicity, gender, respiratory support and fractional concentration of inspired oxygen (FiO_2_) on postnatal days 7, 14, 21 and 28.

Several studies included chest radiographs as a component of the prediction scoring system for BPD. Chest radiographs are a daily routine examination that is part of intensive care for preterm infants, especially during the early postnatal period. In a study, the day 7 chest radiographs with cystic elements or interstitial changes were associated with oxygen dependency at day 28 [10]. Greenough et al. [11] suggested that the day 7 chest radiographs with interstitial shadows or cystic elements predict BPD or death before discharge. Similarly, in our experience, interstitial pneumonia patterns on chest radiographs around the end of the first week of life typically heralded later development of BPD. Furthermore, interstitial pneumonia patterns on the day 7 chest radiographs seem to be associated with histologic chorioamnionitis. The purpose of the present study was to evaluate whether interstitial pneumonia patterns on the day 7 chest radiographs can predict BPD or death and are associated with histologic chorioamnionitis.

## Methods

### Subjects and data collection

This study included all VLBW (<1500 g) infants who were born before 32 gestational weeks and admitted to the neonatal intensive care unit (NICU) at Seoul National University Bundang Hospital between January 2008 and December 2014. Infants who were transferred to other hospitals before 36 weeks PMA and those who died within 7 days of birth were excluded.

Antenatal, perinatal, and neonatal data were retrospectively collected from the original medical records of the infants included in this study. The primary outcome was BPD or death before 36 weeks PMA. BPD was defined as the need for supplemental oxygen or assisted ventilation, including nasal continuous positive airway pressure at 36 weeks PMA [12]. Histologic chorioamnionitis was diagnosed by the presence of neutrophils infiltrating any of the amnion, choriodecidua, umbilical cord, or chorionic plate according to the grading system suggested by Salafia et al. [13]. Respiratory distress syndrome (RDS) was defined by the presence of respiratory distress, as indicated by an increased oxygen requirement (fractional concentration of inspired oxygen ≥0.4) and compatible chest radiologic findings. PDA was diagnosed with echocardiography, and symptomatic PDA was defined as hemodynamically significant PDA requiring medical or surgical closure. Early-onset sepsis was defined as blood culture-proven bacterial sepsis that occurred before 7 days of life while late-onset after 7 days of life.

### Radiologic evaluation for interstitial pneumonia patterns

**Fig. 1:**
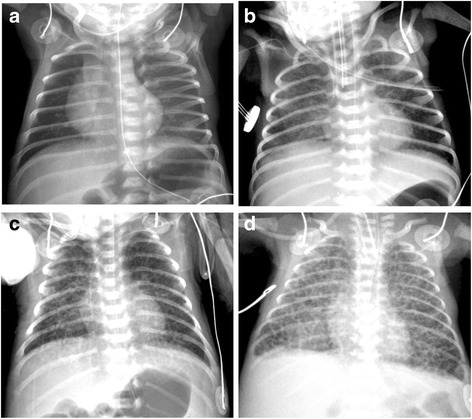
Chest radiographs representing each grade. **a** No demonstrable abnormality (grade 1). **b** Granular infiltration (grade 2). **c** Diffuse streaky interstitial thickening (grade 3). **d** Diffuse coarse interstitial thickening (grade 4)

Chest radiographs obtained on day 7 of life were individually reviewed by two pediatric radiologists (Kim JY, Yun BL) who were unaware of the clinical information regarding each individual infant. Interstitial changes on the day 7 chest radiographs were independently graded by each radiologist with a single grading system (Table 1). Of the total subject preterm infants, chest radiograph images were not obtained from four infants on day 7 of life. As an alternative, chest radiographs on day 6 of life were reviewed for three infants, and a chest radiograph on day 8 of life was reviewed for one infant. Grade 3 and 4 interstitial changes were defined as interstitial pneumonia patterns (Fig. 1).

**Table 1:** Chest radiograph grading system for interstitial changes

Grade	Descriptor
1	No demonstrable abnormality
2	Granular infiltration
3	Diffuse streaky interstitial infiltration
4	Diffuse coarse reticular infiltration

### Statistical analysis

In the univariate analysis, comparisons between groups were performed using the t-test for normally distributed continuous variables, the Mann-Whitney U test for non-normally distributed variables, and the chi-square test for comparison of categorical variables. Multivariate analysis was performed by logistic regression and adjusted odds ratios (OR), and 95% confidence intervals (CI) were calculated. The inter-observer reliability between the two radiologists was tested using kappa (κ) statistics.

Statistical analyses were performed using the IBM SPSS Statistics software package, version 22.0 (IBM, Armonk, NY). Statistical significance was defined as *p* < 0.05.

### Ethical statement

The Seoul National University Bundang Hospital Institutional Review Board (IRB) approved the collection and use of the clinical information for research purposes before the investigation was started and waived the requirement for informed consent (IRB No. B1512–328-105).

## Results

### Clinical characteristics of the subjects

**Fig. 2:**
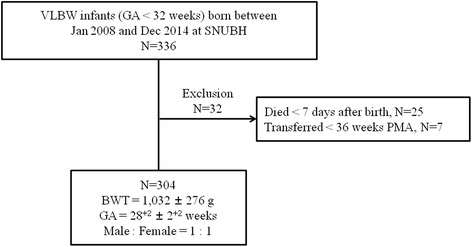
Study subject. VLBW = very low birth weight; GA = gestational age; SNUBH = Seoul National University Bundang Hospital; PMA = postmenstrual age; BWT = birth weight

During the study period, 336 VLBW infants were born before 32 gestational weeks and admitted to our NICU. Among them, 32 infants were excluded: 25 infants who died before 7 days of life and 7 infants who were transferred to other hospitals before 36 weeks PMA. In total, 304 infants were included in the analyses (Fig. 2).

Clinical characteristics of the 304 infants are presented in Additional file 1. The mean gestational age was 28^+2^ weeks (range: 23^+1^–31^+6^ weeks), and the mean birth weight was 1032 g (range: 420–1495 g). The male-to-female ratio of the subjects was 1:1.

### Factors associated with bronchopulmonary dysplasia or death

**Fig. 3:**
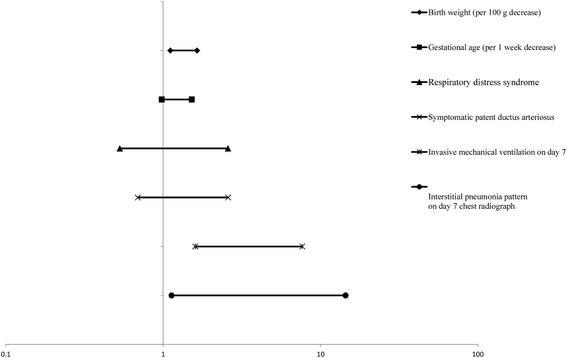
Multivariate modeling with adjusted odds ratios for BPD or death before 36 weeks postmenstrual age

Of the 304 infants, 123 (40.5%) infants developed BPD or died before 36 weeks PMA: 110 infants developed BPD; 13 infants died before 36 weeks PMA. Lower birth weight and gestational age, RDS, symptomatic PDA, invasive ventilation on day 7 of life, and the interstitial pneumonia pattern on the day 7 chest radiograph, which was our primary concern, were factors significantly associated with BPD or death before 36 weeks PMA (Table 2). Multivariate analysis with these significant associated factors included in the logistic regression model revealed that lower birth weight, invasive ventilation on day 7 of life, and the interstitial pneumonia pattern on the day 7 chest radiograph were independently associated with BPD or death before 36 weeks PMA. The adjusted OR and its 95% CI of the interstitial pneumonia pattern on the day 7 chest radiograph for BPD or death before 36 weeks PMA were 4.0 and 1.1–14.4, respectively (Fig. 3). The sensitivity, specificity, positive predictive value, and negative predictive value of the interstitial pneumonia pattern on the day 7 chest radiograph for BPD or death before 36 weeks PMA were 25%, 98%, 89% and 66%, respectively. The specificity and positive predictive value of the interstitial pneumonia pattern on the day 7 chest radiograph for BPD or death was higher than those of birth weight (cut-off value of 1000 g), gestational age (cut-off value of 28 weeks), and invasive ventilation on day 7 of life (Table 3). Comorbidities and outcomes of infants with BPD or death before 36 weeks PMA were presented in Additional file 1.

**Table 2:** Demographic and perinatal characteristics of infants with bronchopulmonary dysplasia or death before 36 weeks postmenstrual age

	No BPD or death *N* = 181	BPD or death *N* = 123	*P*
Birth weight (g), mean ± SD	1161 ± 227	842 ± 228	<0.001
Gestational age (weeks), mean ± SD	29^+1^ ± 1^+5^	26^+6^ ± 1^+6^	<0.001
Male (%)	86 (47.5)	69 (56.1)	0.142
Caesarean section (%)	136 (75.1)	80 (65.0)	0.071
Multiple gestation (%)	54 (29.8)	40 (32.5)	0.616
Antenatal steroid (%)	165 (91.2)	106 (86.2)	0.191
Premature rupture of membrane (%)	80 (44.2)	54 (43.9)	1.000
Preeclampsia (%)	49 (27.1)	29 (23.6)	0.507
Histologic chorioamnionitis (%)	86 (47.5)	59 (48.0)	1.000
Respiratory distress syndrome (%)	101 (55.8)	107 (87.0)	<0.001
Symptomatic patent ductus arteriosus (%)	74 (40.9)	91 (74.0)	<0.001
Early-onset neonatal sepsis (%)	1 (0.6)	2 (1.6)	0.568
Invasive ventilation on day 7 of life (%)	16 (8.8)	74 (60.2)	<0.001
Interstitial pneumonia patterns on day 7 chest radiographs (%)	4 (2.2)	31 (25.2)	<0.001

*BPD* bronchopulmonary dysplasia, *SD* standard deviation

**Table 3:** Predictive characteristics of interstitial pneumonia patterns on day 7 chest radiographs and other associated factors for BPD or death before 36 weeks postmenstrual age

	Cut-off value^a^	Sensitivity	Specificity	PPV	NPV
Birth weight	1000 g	66%	84%	74%	75%
Gestational age	28 weeks	80%	72%	66%	84%
Invasive ventilation on day 7 of life	-	60%	91%	82%	77%
Interstitial pneumonia patterns on day 7 chest radiographs	-	25%	98%	89%	66%

*PPV* positive predictive value, *NPV* negative predictive value

^a^ Derived from receiver operating characteristic curve

### Interstitial pneumonia pattern on the day 7 chest radiograph

Of the 304 infants, 35 (11.5%) infants exhibited the interstitial pneumonia pattern on the day 7 chest radiographs. Agreement between the two radiologists in grading interstitial changes on the day 7 chest radiographs had good inter-observer reproducibility (*r* = 0.940, *p* < 0.001). Especially for grade 3 and 4 interstitial changes, which were used to define the interstitial pneumonia pattern, the agreement between the two radiologists was 100%.

### Factors associated with the interstitial pneumonia pattern on the day 7 chest radiograph

**Fig. 4:**
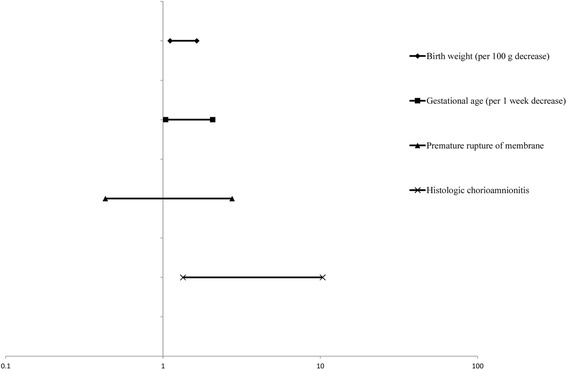
Multivariate modeling with adjusted odds ratios for the interstitial pneumonia pattern on the day 7 chest radiograph

Lower birth weight and gestational age, RDS, premature rupture of membrane, and histologic chorioamnionitis were factors significantly associated with the interstitial pneumonia pattern on the day 7 chest radiograph (Table 4). Multivariate analysis with these significantly associated factors included in the logistic regression model revealed that lower birth weight and gestational age and histological chorioamnionitis were independently associated with the interstitial pneumonia pattern on the day 7 chest radiograph. The adjusted OR and its 95% CI of histologic chorioamnionitis for the interstitial pneumonia pattern on the day 7 chest radiograph were 3.7 and 1.3–10.3, respectively (Fig. 4).

**Table 4:** The perinatal factors associated with interstitial pneumonia patterns on day 7 chest radiographs

	No interstitial pneumonia patterns on day 7 *N* = 269	Interstitial pneumonia patterns on day 7 *N* = 35	*P*
Birth weight (g), mean ± SD	1062 ± 267	793 ± 220	<0.001
Gestational age (weeks), mean ± SD	28^+3^ ± 1^+6^	25^+6^ ± 1^+4^	<0.001
Male (%)	138 (51.3)	17 (48.6)	0.858
Caesarean section (%)	194 (72.1)	22 (62.9)	0.321
Multiple gestation (%)	84 (31.2)	10 (28.6)	0.847
Antenatal steroid (%)	242 (90.0)	29 (82.9)	0.243
Premature rupture of membrane (%)	113 (42.0)	21 (60.0)	0.048
Preeclampsia (%)	72 (26.8)	6 (17.1)	0.303
Histologic chorioamnionitis (%)	118 (43.9)	27 (77.1)	<0.001

## Discussion

To date, not a few studies have suggested chest radiograph scoring systems to predict BPD [10, 11, 14–19]. However, the currently described scoring systems are often too complex for clinicians to use at the bedside [10, 11, 14] or are applied to chest radiographs taken at 3–4 weeks of age when BPD is already being established [15]. High-resolution chest computed tomography (CT) has been used in recent studies to evaluate the severity of BPD in older-aged infants [20]. However, it is difficult to apply chest CT in practice to newborn preterm infants for the early prediction of BPD. By contrast, our chest radiograph grading system is very simple in that it only evaluates interstitial changes and consists of only four grades. The ease and clarity of our grading system are supported by the fact that there was a high degree of agreement in the grading results between the two radiologists. Our grading system also uses chest radiographs taken on day 7 of life, which is a time point when whether the preterm infants will progress to BPD or not cannot be judged easily. In this regard, our chest radiograph grading system for the prediction of BPD has advantages over other scoring systems using chest radiographs and is expected to be readily available to clinicians at the bedside.

The present study showed that the interstitial pneumonia pattern on the day 7 chest radiograph was significantly associated with BPD or death before 36 weeks PMA and predicted BPD or death with 98% specificity in VLBW infants who were born before 32 weeks PMA. Accordingly, the interstitial pneumonia pattern on the day 7 chest radiograph can help clinicians identify high-risk group of infants for BPD or death at a relative early postnatal age.

Early identification of high-risk infants for BPD is crucial to implement potentially preventive or early therapeutic interventions for BPD. Aggressive application of non-invasive ventilation, more restrictive use of oxygen, and postnatal corticosteroids or novel therapies that are currently being evaluated, including stem cells, would be more effective when implemented as early as possible before structural damage is solidified [21–26]. The previously suggested prediction models for BPD include birth weight, gestational age, gender, and duration of oxygen therapy and mechanical ventilation as the major risk factors [8, 9, 27–29]. Similarly, birth weight, gestational age, and invasivel ventilation on day 7 of life were significant risk factors for BPD or death in our study, and their effects on BPD or death were adjusted during the multivariate analysis step.

As mentioned above, chest radiographs have also been tested for predictors for BPD [10, 11, 14–19]. Several chest radiographic scoring systems have been suggested as prediction tools for BPD. Yuksel et al. showed that radiographic appearance at 7 days of life reached 71% sensitivity and 88% specificity in predicting BPD [10]. Chest radiographs were scored according to the volume of the thorax, presence of opacification, haziness, interstitial changes and cystic elements. Greenough et al. [11] suggested that chest radiographs at 7 days of life can be used to predict BPD or death before discharge. They found that the development of BPD can be predicted by the presence of interstitial shadows or fibrosis, whereas death can be predicted by the presence of cysts. In our study, we focused on the interstitial changes alone in evaluating chest radiographs. The interstitial changes on the day 7 chest radiographs were easily noticeable, and a high degree of agreement between observers was obtained [16]. Furthermore, interstitial changes on chest radiograph are less influenced by inflation status, which will be variable in the preterm infants. Interstitial changes on chest radiograph indicate lung injury, including airway and pulmonary vascular damages and interstitial edema [17]. The presence of interstitial changes on chest radiograph is significantly associated with BPD [10, 11, 18] or adverse respiratory outcomes [19]. Cystic changes on chest radiograph are also associated with BPD or death [11]. However, we did not evaluate cystic changes in this study because cystic changes on chest radiographs were more difficult to distinguish from other diseases, such as infection, sequelae of pulmonary hemorrhage, and pulmonary interstitial emphysema [16]. In addition, cystic changes on chest radiograph represent chronic pathological changes and appear later [17].

Of note, interstitial changes appear from as early as the first several days of life. By day 7 of life, interstitial changes became obvious in preterm infants with the interstitial pneumonia patterns on their chest radiographs. With regard to predicting BPD using chest radiographic findings, the subjectivity of the reviewers and lack of inter-observer agreement have been reported to be problematic [30]. In the present study, we used a simple chest radiographic grading system that exclusively assessed interstitial changes, and good inter-observer reproducibility was obtained between two radiologists. Taken together, the interstitial pneumonia pattern on the day 7 chest radiograph may be an early and objective predictor of BPD or death that can be utilized to select preterm infants at high risk of BPD or death as early as possible.

Whereas the interstitial pneumonia pattern on the day 7 chest radiograph was associated with future BPD or death, it was also associated with histologic chorioamnionitis in the past. The association of the interstitial pneumonia pattern on the day 7 chest radiograph with previous histologic chorioamnionitis remained significant after adjustment for birth weight, gestational age, and premature rupture of membrane. Several studies have demonstrated that chorioamnionitis or intrauterine infection can disrupt lung development by inducing fetal pulmonary inflammatory responses [31–35]. A meta-analysis also demonstrated that histologic chorioamnionitis is significantly associated with the increased incidence of BPD [36]. In this regard, the interstitial pneumonia pattern on the day 7 chest radiographs might correspond to a phase in the pathophysiology of pulmonary inflammation that starts with chorioamnionitis and leads to a later diagnosis of BPD. The most frequently discovered microorganism in the amniotic fluid of pregnant woman with histologic chorioamnionitis is *Ureaplasma urealyticum*, which is a proven cause of fetal and neonatal pneumonia [37]. Thus, the interstitial pneumonia pattern on the day 7 chest radiograph may be a reflection of interstitial pneumonia caused by microorganisms, including *Ureaplasma urealyticum*. However, interstitial thickening due to interstitial hypercellularity and focal deposition of elastin and collagen are common pathologic changes revealed in the evolving BPD [38]. The interstitial pneumonia pattern may also be a reflection of these pathologic changes. At this point, it is not clear whether the interstitial pneumonia pattern is a reflection of interstitial pneumonia caused by microorganisms or a reflection of early pathologic changes observed in evolving BPD.

The main limitation of the present study is the retrospective design from a single center. Further prospective studies are required to validate our results. In addition, further research is needed to clarify the underlying pathology reflected by the interstitial pneumonia pattern on the chest radiograph in preterm infants who later develop BPD.

## Conclusions

The findings of this study suggest that the interstitial pneumonia pattern on the day 7 chest radiograph predicts BPD or death before 36 weeks PMA. Chest radiographs are used in daily clinical practice, and the interstitial pneumonia pattern on the day 7 chest radiograph is easy to identify. Furthermore, the interstitial pneumonia pattern on the day 7 day chest radiograph has a high specificity for predicting BPD or death and can be utilized to select high-risk preterm infants who will benefit most from potentially preventive interventions against BPD.

## Additional files

Additional file 1: Perinatal and neonatal clinical characteristics of study subject. (DOCX 16 kb)

Additional file 2: Raw data. (XLSX 45 kb)

## Abbreviations

BPD: Bronchopulmonary dysplasia
NICU: Neonatal intensive care unit
PDA: Patent ductus arteriosus
PMA: Postmenstrual age
RDS: Respiratory distress syndrome
VLBW: Very low birth weight
