# Supplementary material for: Interstitial pneumonia pattern on day 7 chest radiograph predicts bronchopulmonary dysplasia in preterm infants
Source: BMC Pediatr. 2017 May 15;17:125. doi: 10.1186/s12887-017-0881-1 (PMC5433188; doi:10.1186/s12887-017-0881-1)
Supplement: Supplementary file 1 — Perinatal and neonatal clinical characteristics of study subject. (DOCX 16 kb) [file 12887_2017_881_MOESM1_ESM.docx]

Additional file 1. Perinatal and neonatal clinical characteristics of study subject

|  | N=304 |
| --- | --- |
| Birth weight (g), mean±SD (range) | 1,032 ± 276 (420 – 1,495) |
| Gestational age (weeks), mean±SD (range) | 28^+2^ ± 2^+2^ (23^+1^ – 31^+6^) |
| Male (%) | 155 (51.0) |
| Caesarean section (%) | 216 (71.1) |
| Multiple gestation (%) | 94 (30.9) |
| Antenatal steroid (%) | 271 (89.1) |
| Premature rupture of membrane (%) | 134 (44.1) |
| Preeclampsia (%) | 78 (25.7) |
| Histologic chorioamnionitis (%) | 145 (47.7) |
| Apgar score at 1 min, mean±SD (range) | 4.2 ± 2.0 (0 – 9) |
| Apgar score at 5 min, mean±SD (range) | 6.3 ± 1.8 (1-10) |
| Respiratory distress syndrome (%) | 208 (68.2) |
| Symptomatic patent ductus arteriosus (%) | 165 (54.3) |
| Early-onset neonatal sepsis (%) | 3 (1.0) |
| Late-onset neonatal sepsis (%) | 30 (9.9) |
| Interstitial pneumonia patterns on day 7  chest radiographs | 35 (11.5) |
| Invasive mechanical ventilation on day 7 | 90 (29.6) |
| BPD or death before 36 weeks PMA (%) | 123 (40.5) |
| BPD (%) | 110 (37.8*) |
| Death before 36 weeks PMA (%) | 13 (4.3) |
| Death during initial admission (%) | 15 (4.9) |
| Necrotizing enterocolitis (Bell’s stage ≥2) (%) | 24 (7.9) |
| Intraventricular hemorrhage (%) | 63 (20.7) |
| LASER for retinopathy of prematurity (%) | 65 (21.4) |
| Duration of invasive mechanical ventilation (days), mean±SD (range) | 16.4 ± 41.1 (0 – 376) |
| Length of hospital stay (days), mean±SD (range) | 82.5 ± 61.2 (8 – 570) |

* 13 infants who died before 36 weeks PMA were excluded.

SD = standard deviation; BPD = bronchopulmonary dysplasia; PMA = postmenstrual age

Supplement 2. Comorbidities and outcomes of infants with bronchopulmonary dysplasia or death before 36 weeks postmenstrual age

|  | No BPD or death  N=181 | BPD or death  N=123 | *P* |
| --- | --- | --- | --- |
| Late-onset neonatal sepsis (%) | 8 (4.4) | 22 (17.9) | <0.001 |
| Necrotizing enterocolitis  (Bell’s stage ≥2) (%) | 10 (5.5) | 14 (11.4) | 0.082 |
| Intraventricular hemorrhage (%) | 21 (11.6) | 42 (34.1) | <0.001 |
| LASER for retinopathy of prematurity (%) | 22 (12.1) | 43 (35.0) | <0.001 |
| Duration of invasive mechanical ventilation (days), mean±SD | 2.9 ± 5.4 | 36.2 ± 59.0 | <0.001 |
| Length of hospital stay (days), mean±SD | 62.4 ± 21.5 | 112.0 ± 84.4 | <0.001 |
| Death during initial admission (%) | 0 (0.0%) | 15 (12.2%) | <0.001 |

BPD = bronchopulmonary dysplasia; SD = standard deviation
